# Medication Adherence and Contributing Factors in Patients with Heart Failure Within the Middle East: A Systematic Review

**DOI:** 10.5334/gh.1431

**Published:** 2025-05-27

**Authors:** Mohammed Alfaqeeh, Ramez Abdullah, Neily Zakiyah, Auliya A. Suwantika, Maarten J. Postma, Fita Rahmawati, Anna Wahyuni Widayanti, Baharudin Ibrahim

**Affiliations:** 1 Department of Pharmacology and Clinical Pharmacy, Faculty of Pharmacy, Universitas Padjadjaran, Bandung, Indonesia; 2 Department of Pharmacology and Clinical Pharmacy, Faculty of Pharmacy, Universitas Gadjah Mada, Yogyakarta, Indonesia; 3 Center of Excellence for Pharmaceutical Care Innovation, Universitas Padjadjaran, Bandung, Indonesia; 4 Center for Health Technology Assessment, Universitas Padjadjaran, Bandung, Indonesia; 5 Department of Health Sciences, University Medical Center Groningen, University of Groningen, Groningen, The Netherlands; 6 Department of Economics, Econometrics and Finance, Faculty of Economics and Business, University of Groningen, Groningen, The Netherlands; 7 Department of Pharmaceutics, Faculty of Pharmacy, Universitas Gadjah Mada, Yogyakarta, Indonesia; 8 Department of Clinical Pharmacy and Pharmacy Practices, Faculty of Pharmacy, Universiti Malaya, Kuala Lumpur, Malaysia

**Keywords:** Heart failure, Medication adherence, non-adherence factors, Quality assessment

## Abstract

Medication adherence among heart failure (HF) patients in the Middle East remains a critical, under-explored issue. This systematic review examines medication adherence rates and their influencing factors in this region. A search of PubMed, Scopus, and Google Scholar identified 12 studies published between 2013 and 2023, revealing an average non-adherence rate of 60%. Influential factors include socioeconomic status, education, psychiatric conditions, and medication-related issues. Significant gaps in research methodologies and reporting were noted. The review highlights the impact of socio-economic determinants on adherence and calls for more robust research and targeted interventions to address these barriers. Improving patient education and healthcare provider communication are crucial to enhancing adherence rates. These findings underscore the importance of addressing regional challenges through tailored approaches and suggest further studies are needed to develop effective strategies for improving adherence among HF patients in the Middle East.

## 1. Introduction

Heart failure (HF) is characterized as a pathological state where the heart cannot pump blood effectively due to structural or functional cardiac abnormalities, impairing its ability to fill or eject blood (1). Factors contributing to HF include myocardial infarctions, progressive cardiac deterioration, or genetic defects (2,3). This condition, often resulting from left ventricular, right ventricular, or biventricular dysfunction, may also involve complications in the endocardium, myocardium, pericardium, major vessels, or heart valves, highlighting its complex etiology (4,5). Pathogenic processes, such as hemodynamic overload, ventricular remodeling, and ischemic dysfunction, play key roles in its development, illustrating HF’s multifactorial nature (6).

The global burden of HF is substantial, with its prevalence exerting significant health and economic impacts across both developed and developing countries, which may reduce quality of life and increase rates of hospital readmissions, leading to high morbidity and mortality rates (5,7,8). In the United States (US), HF affects approximately 6.5 million adults (9). This prevalence is not isolated to the US; similar prevalence rates were observed in Australia and Canada, where the incidence of HF also falls within 1% to 2% of the general population (4,10). However, the burden of HF in the Middle East is exceptionally pressing. Studies indicate that the prevalence of HF in Middle Eastern countries is rising, with an estimated 3.75 million patients, driven by high rates of risk factors such as hypertension, diabetes, and ischemic heart disease, often occurring at a younger age compared to Western populations. This early onset contributes to a higher lifetime risk of HF and an increased economic burden on the healthcare systems in these countries (7,11,12).

In addition to the clinical and economic burdens, medication adherence is a notable issue in the Middle East, with non-adherence rates ranging from 1.4% to 88%, depending on the country and condition (7,11,12). Contributing factors include cultural beliefs that may lead to skepticism about modern medicine, variability in healthcare system quality, economic constraints such as medication costs and insurance coverage, and gaps in patient education regarding the importance of following prescribed treatments. Studies, including those from Saudi Arabia and Jordan, have identified forgetfulness, side effects, and misunderstanding of medication regimens as major issues impacting adherence. Addressing these challenges requires a comprehensive approach that takes into account the region’s socioeconomic and cultural context, including educational programs, enhanced communication between patients and healthcare providers, and the use of adherence aids and technologies (13,14,15).

These statistics are alarming, with Rizzuto et al. (16) reporting that approximately 25% of HF patients face readmission within a month of discharge and Desai and Stevenson (17) noting that half are readmitted within six months. Nonadherence to treatment plans is one of the key contributing factors to these high readmission rates. This cyclical pattern of hospitalization highlights the severity of HF and the considerable economic impact, with Heidenreich et al. (18) projecting the cost of HF care in the US to potentially reach $70 billion annually in the next decade.

The statistics are concerning, with a previous study indicating that approximately 25% of HF patients are readmitted within the first month after discharge (16), while another study reports that nearly half of these patients experience readmission within six months (17). Nonadherence to treatment plans is one of the key contributing factors to these high readmission rates. This persistent cycle of hospitalization not only underscores the severity of HF but also highlights its substantial economic impact, with projections estimating that the cost of HF care in the United States could escalate to $70 billion annually within the next decade (18). Therefore, optimal management of HF necessitates a focus on patient behavior, especially regarding medication adherence, a factor critical to improving treatment efficacy, patient well-being, and reducing healthcare expenditure (19). The World Health Organization (WHO) (20) stresses the significance of maintaining regular medication adherence when managing chronic diseases, highlighting its role in minimizing comorbidities, hospitalizations, and mortality. A study revealed that patients’ perceptions of their symptoms could influence their adherence to medication regimens, indicating the nuanced nature of compliance in HF (21). Another study reported variability in adherence rates among HF patients, ranging from 10% to 93%, with a majority achieving 40% to 60% adherence (22). This variability, alongside the challenges of integrating pharmacological and lifestyle interventions like diet and exercise, particularly among older adults (23), highlights the imperative for precise strategies to improve adherence. Addressing these complexities is essential for enhancing HF management outcomes.

Medication adherence is influenced by a complex interplay of factors across five key domains: patient characteristics, socioeconomic status, healthcare system dynamics, therapy-related issues, and condition-specific elements (20). A comprehensive analysis identified over 771 factors affecting adherence to long-term treatment (24). Most of the identified factors influenced the initial behavior of starting adherence (implementation), while only a small number of factors specifically influenced the ability to maintain long-term adherence (persistence). Effective management requires understanding these diverse factors to tailor strategies enhancing adherence. Yet, despite the extensive research conducted globally on the adherence to medication among patients with HF, including systematic reviews that have identified various factors associated with adherence (22,25,26,27), there is a lack of comprehensive systematic reviews investigating these aspects within the context of Middle Eastern countries. Thus, this systematic review aims to examine medication adherence rates and the determinants influencing adherence among patients suffering from HF in Middle Eastern countries.

## 2. Methods

This systematic review was meticulously formulated following the guidelines outlined in the Preferred Reporting Items for Systematic Reviews and Meta-Analyses (PRISMA) (28).

### 2.1 Data search strategy

A systematic literature search was conducted on three major databases: Scopus, PubMed, and Google Scholar. The search methodology involved utilizing a blend of keywords and Boolean operators to pinpoint studies centered on medication compliance among individuals suffering from HF in Middle Eastern countries. The detailed search strategy utilized across these databases is summarized in supplementary materials Table S1. The search scope was refined to include studies published between 2013 and 2023. The selected period covers the past decade to capture advancements in medication adherence research for HF patients in the Middle East, reflecting current medical practices, healthcare systems, and patient behaviors. Moreover, manual searches were conducted on the reference lists of identified articles and systematic reviews to uncover additional pertinent studies. This search strategy was designed to capture a comprehensive range of literature addressing medication adherence in the context of HF patients.

### 2.2 Study selection and eligibility requirements

This systematic review includes primary research studies targeting adults over 18 years of age diagnosed with HF. The scope is confined to peer-reviewed or original articles that delve into the factors influencing medication adherence and its measurement. Studies were conducted in Middle Eastern countries, with the publication period between 2013 and 2023. The review focuses on studies from 2013 onward to capture the most recent advancements in healthcare, treatment protocols, and medication adherence strategies in HF patients. It is essential that these studies explicitly describe their adherence measurement methodologies. Eligible publications must be in English or Arabic, clearly delineating the sample size and research setting. Exclusion criteria included studies that primarily address a broader spectrum of cardiovascular diseases, except where patients with HF constitute more than 50% of the study’s population. Also, studies that merely report adherence levels do not investigate underlying factors, and research focuses on patients with multimorbidity. The study selection process was managed using Mendeley 2.119.0 software to organize the collected records. We first removed duplicates through both automated and manual methods. Afterward, we screened the titles and abstracts of the remaining studies to assess their alignment with the inclusion criteria. Full texts of studies that appeared relevant were then reviewed. The selection process was carried out by two researchers (MA and RA), and any disagreements were resolved through discussions that included all researchers (MA, RA, NZ, AAS, MJP, FR, AWW, and BI).

### 2.3 Outcome measures

The primary objective of this review was to evaluate the degree of medication adherence and non-adherence among individuals with HF in Middle Eastern countries, represented through the overall participant percentage. The secondary outcome covered the determinants that affect medication adherence through an analysis of quantitative data from the studies reviewed, regardless of the reported importance of these factors. This approach was intended to offer a detailed examination of adherence issues in the Middle East.

### 2.4 Quality evaluation

The quality evaluation utilized the Crowe Critical Appraisal Tool (CCAT) (29,30). Derived from a comprehensive collection of prior critical appraisal instruments, this instrument is rooted in established research methodology theories and reporting standards. Its credibility and accuracy have been verified through rigorous testing, with Crowe and associates revealing a consistency of 0.83 and an absolute agreement of 0.74 (30).

The tool is designed for independent scoring, with a maximum achievable score of 40, and is adaptable to various research formats, including observational and experimental studies. Structured into 22 items across eight categories, each item offers descriptors for evaluation and scoring. Scores range from 0 to 5 on a six-point scale, where 0 represents the lowest attainable score, and 5 reflects the highest possible score. Each study underwent a quality assessment process, resulting in the calculation of a percentage-based score as per the guidelines outlined in the CCAT User Guide. Two reviewers, MA and RA, conducted independent assessments of each study. Subsequently, the reviewers deliberated on the CCAT scores until a consensus was reached.

### 2.5 Data synthesis and analysis

Following the guidelines stipulated by PRISMA, our systematic review undertook a meticulous approach to ensure transparency and replicability of our findings on medication adherence among HF patients in the Middle East. The completed PRISMA checklist was provided as supplementary material.

We have designed and employed a comprehensive data extraction tool to achieve the required data synthesis. This piloting process helps refine the tool to capture all relevant information effectively and to systematically collect critical information from each study, including the authors, country, publication year, setting, adherence definition, objectives, sample size, design, inclusion and exclusion criteria, participant demographics, rates of non-adherence, methods of adherence measurement, factors affecting adherence, study conclusions, limitations, and the Crowe Critical Appraisal Tool (CCAT) score for methodological quality assessment. This rigorous extraction process was conducted by two independent reviewers, MA and RA, with any discrepancies resolved through consensus, ensuring a thorough and unbiased evaluation of the literature. Including qualitative and quantitative studies allowed for a detailed narrative synthesis, conforming to recommendations for systematic reviews with varied study designs. Through qualitative narrative synthesis, we explored the complexities of medication adherence, identifying common themes and unique insights into the factors affecting adherence among HF patients in the Middle East.

## 3. Results

**Figure 1:**
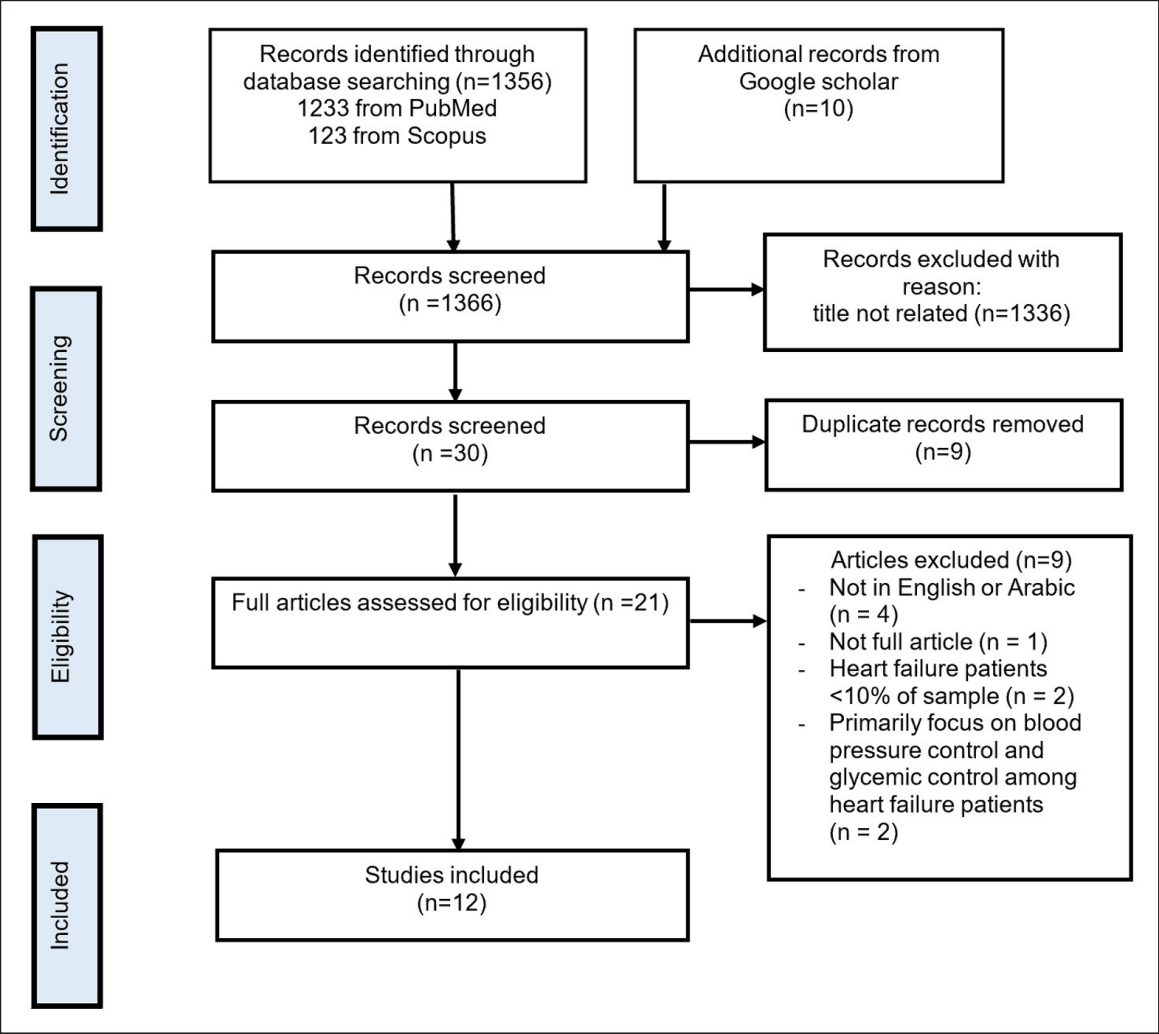
Flowchart for studies included according to PRISMA criteria.

The systematic review commenced by identifying 1,356 records through database searches, with 1,233 sourced from PubMed, 123 from Scopus, and 10 from Google Scholar, resulting in a total of 1,366 records screened. Figure 1 illustrates the flow diagram for article selection, providing a visual representation of the screening process. Following the screening process, 1,336 records were eliminated because their titles and abstracts did not meet the study’s criteria. Subsequently, 30 records underwent further screening, during which nine duplicate records were identified and removed. This left 21 full articles for eligibility assessment. Among these, nine articles were excluded for various reasons: four were not available in English or Arabic, one lacked full article format, two had HF patient representations of less than 10% in the sample, and two primarily focused on blood pressure and glycemic control among HF patients. Ultimately, 12 studies fulfilled the inclusion criteria and were incorporated into the review (31,32,33,34,35,36,37,38,39,40,41,42).

### 3.1. Geographical patterns and patient demographics

The selected studies exclusively utilized quantitative methodologies with a cross-sectional design. Iran emerges as the most prominent focus, with eight studies (32,33,36,37,39,40,41,42). Jordan follows with two studies (34,35), while Saudi Arabia and Yemen are the subjects of one study each within the same timeframe (31,38). All studies identified had a quantitative nature; no qualitative studies were found in this review. Notably, all studies targeted HF patients, except for the studies that specifically examined congestive HF patients (31,38).

The review collated data from a cumulative sample size of 3,158 participants, with a majority of 56% (1,764 individuals) being male and the remaining 44.15% (1,394 individuals) being female. A notable demographic finding was the identification of illiteracy in 585 participants, constituting almost 20% of the sample size, suggesting potential challenges in patient education and treatment adherence.

### 3.2. Medication adherence

This review reveals a significant variance in medication non-adherence rates among HF patients in Middle Eastern countries, with figures influenced by both geographic and methodological factors. Countries such as Jordan, Saudi Arabia, Yemen, and Iran report high levels of non-adherence, but the exact rates vary considerably depending on the adherence measurement tool used. For example, differences between instruments such as the General Medication Adherence Scale (GMAS), the 4-item and 8-item versions of the Morisky Medication Adherence Scale (MMAS-4 and MMAS-8), the 10-item Medication Adherence Report Scale (MARS-10), and the Modanloo questionnaire contribute to this wide range. These discrepancies emphasize the complexity of measuring adherence and the influence of contextual factors such as patient behavior, healthcare access, and health system characteristics. Detailed adherence rates by country and measurement tool are presented in Table 1.

**Table 1:** Summary of the included studies.


AUTHORS/ SETTING/ COUNTRY	SAMPLES	METHODS	ADHERENCE MEASURE	DURATION OF DISEASE	AVERAGE AGE (YEARS)	LEVEL OF EDUCATION	NON-ADHERENCE %	CCAT %

Jarrah et al. 2023 (35) Outpatient clinic Jordan	164	Cross-sectional study	General Medication Adherence Scale (GMAS)	Not indicated	62.5 ± 8.8	The majority (53%) are less than or equal to high school	47.00%	83%

Jarab et al. 2023 (34) Outpatient clinic Jordan	427	Cross-sectional study	Morisky (MMAS-4)	6.41 ± 6.16	63 ± 12	The majority (69.4%) are not complete their educational pursuits	92.50%	80%

Shojaee et al. 2023 (42) Outpatient clinic Iran	474	Cross-sectional study	Modanloo questionnaire	62.4% less 6 years HF	52.86 ± 15.20	0% illiterate, and 44.5% upper diploma	49.30%	80%

Moaddab et al. 2023 (37) NI Iran	115	Cross-sectional study	Medication adherence report scale (MARS-5)	Not indicated	64.01 ± 11.46	53.91% illiterate	Not indicated	78%

Sadeghiazar et al. 2022 (40) Hospitalized patient Iran	237	Cross-sectional study	The Medication Adherence Report Scale (MARS-10)	Majority 63% less than 9 years	64.81 ± 14.00	49.45% illiterate	76.90%	80%

Amininasab et al. 2018 (32) Hospitalized patient Iran	300	Cross-sectional study	Morisky (MMAS-8)	Not indicated	64.15 ± 11.47	31.8% illiterate	60.30%	75%

Rezaei et al. 2022 (39) Outpatient clinic Iran	250	Cross-sectional study	Morisky (MMAS-8)	Majority (71.8%) less than 10 years	The majority (76%) aged 41–80 years	0% illiterate, and (57.6%) diploma and upper	61%	75%

Shamsi et al. 2020 (41) Outpatient clinic Iran	150	Cross-sectional study	Morisky (MMAS-8)	Not indicated	48.48 ± 12.25	The majority (57.33%) are basic to high school	23.33%	70%

Lin et al. 2020 (36) Outpatient clinic Iran	468	A longitudinal study using cross-sectional design	Medication adherence report scale (MARS-5)	5.5 ± 3.6	69.3 ± 7.3	Mean 6.8 ± 2.9 yrs	Not indicated	90%

Amininasab et al. 2017 (33) Hospitalized patient Iran	300	Cross-sectional study	Morisky (MMAS-8)	Not indicated	64.15 ± 11.47	50.3% illiterate	Not indicated	63%

Raffaa et al. 2020 (38) Outpatient clinic Saudi Arabia	151	Cross-sectional study	Morisky (MMAS-4)	47% less than 3 years	The majority (66.2%) aged above 60 years	37.7% illiterate	53.60%	55%

Al-Zaazaai et al. 2019 [31] Hospitalized patient Yemen	86	Cross-sectional study	Morisky (MMAS-4)	Not indicated	56.9 ± 16.0	53% illiterate	56.00%	55%


**Abbreviations:** CCAT: Crowe Critical Appraisal Tool; HF: heart failure; NI: not indicated.

### 3.3. Factors affecting adherence

The factors identified from the included studies were systematically classified into various categories, as illustrated in Table 2. This classification provides a clear overview of the diverse influences on medication adherence among HF patients, facilitating a better understanding of the complex relationships between these factors.

**Table 2:** Influences on medication adherence.


	POSITIVE EFFECT ON ADHERENCE	NEGATIVE EFFECT ON ADHERENCE	NEUTRAL EFFECT ON ADHERENCE

**Patient-related factors**			

Age	n = 1 **Jordan** Younger than 60 years (35)	n = 1 **Iran** Aged 54 years and above (42)	n = 8 **Iran** (32,36,39,40,41) **Yemen** (31) **Saudi Arabia** (38) **Jordan** (34)

Gender		n = 2 **Iran** Female (39,40)	n = 8 **Iran** (32,36,37,41,42) **Yemen** (31) **Saudi Arabia** (38) **Jordan** (35)

Marital status	n = 2 **Saudi Arabia** Married (38) **Jordan** Married (35)	n = 1 **Jordan** Living alone (35)*	n = 3 **Iran** (40,42) **Jordan** (34)

BMI			n = 5 **Iran** (36,40,42) **Jordan** (34,35)

Smoking status		n = 1 **Yemen** Smoking (31)	n = 6 **Iran** (36,39,42) **Saudi Arabia** (38) **Jordan** (34,35)

Physical activity			n = 3 **Iran** (39) **Saudi Arabia** (38) **Jordan** (34)

Family history			n = 1 **Jordan** (34)

Temperament factors			n = 1 **Iran** cyclothymic, anxious, hyperthymic, irritable, and depressive temperament (41)

Housing	n = 1 **Saudi Arabia** Urban residence (38)		n = 3 **Iran** (32,42) **Jordan** Residential circumstances (34)

Alcohol usage			n = 1 **Iran** (39)

Number of children	n = 1 **Iran** Less than 5 children (32)*		

Khat chewing habit		n = 1 **Yemen** Khat use (31)	

Diet control		n = 1 **Saudi Arabia** No diet control (38)*	

Biomedical test			n = 2 **Jordan** (34) **Iran** Blood pressure and heart rate (36)

**Condition-related factor**s			

Duration of HF	n = 2 **Saudi Arabia** Newly Diagnosed (38) **Iran** Diagnosed < 5 years (39)	n = 1 **Iran** Over six years with HF (42)	n = 2 **Jordan** (34) **Iran** (36)

Comorbidities	n = 1 **Iran** Mitral valve regurgitation (42)	n = 1 **Jordan** Diabetes Mellitus (35)*	n = 4 **Saudi Arabia** (38) **Jordan** (34,35) **Iran** (40)

NYHA classification	n = 1 **Iran** Class 1 (42)	n = 1 Iran Higher NYHA class (40)	n = 2 **Jordan** (34) **Iran** (36)

Ejection fraction	n = 1 **Iran** 50–60% (42)	n = 1 **Iran** 30–40% (34)*	n = 3 **Jordan** (34,35) **Iran** (40)

Number of hospitalizations	n = 1 **Jordan** Ever been hospitalized for HF (35)		n = 1 **Iran** (32)

Number of other chronic diseases			n = 1 **Jordan** (34)

Coronary artery bypass graft surgery	n = 1 **Iran** History of CABG (40)		

Psychiatric conditions		n = 5 **Iran** Psychological distress (33,36) Depression (41) Insomnia (36) Lack of peace of mind, dependency and existential distress (33)	

Cognitive function			n = 1 **Iran** Mini Mental State (36)

Sleep disorders		n = 2 **Jordan** Difficulty of sleeping (35)* Iran Insomnia (36)	

**Socioeconomic factors**			

Income level		n = 3 **Saudi Arabia** High income (38) **Jordan** low income (35)* Iran low Income (40)	n = 2 **Iran** (32) **Jordan** (34)

Education level	n = 3 **Iran** Post-secondary education (32,42) **Jordan** Post-secondary education (35)	n = 4 **Yemen** Illiterate (31) **Saudi Arabia** High education level (38) **Jordan** Lower levels of education (34) **Iran** Lower levels of education (40)	n = 2 **Iran** (36,39)

Employment status		n = 1 **Saudi Arabia** Employed (38)*	n = 3 **Iran** (32,40) **Jordan** (34)

Insurance	n = 1 **Jordan** Insured (35)	n = 1 **Yemen** Uninsured (31)	n = 1 **Iran** (42)

Health literacy	n = 1 **Iran** eHealth literacy (36)	n = 1 **Iran** Insufficient health literacy (39)	

Social media	n = 1 **Iran** Familiar with social media (42)		

**Medication factors**			

Treatment duration		n = 1 **Saudi Arabia** long treatment period (38)	

Adverse drug reactions		n = 1 **Jordan** Side effects (34)	

Number of medications	n = 2 **Iran** Consuming less than five tablets daily (32)* Jordan Prescribed ACEIs/ARBs and statin therapy (34)	n = 1 **Jordan** On anticoagulant medication regimen (34)	n = 5 **Iran** Medication type or number of Medication (36,39,40) **Saudi Arabia** Daily medication intake (38) **Jordan** Medication count, dosage intervals, satisfaction with treatment, varieties of HF pharmaceuticals, and additional medicinal treatments (34)

**Institutional factors**			

Caregiver support			n = 1 **Jordan** (35)

HF control health education	n = 1 **Iran** Awareness of HF (42)	n = 2 **Saudi Arabia** Receiving education (38) **Saudi Arabia** Received Pharmaceutical health education (38)	

Distance to Hospital	n = 1 **Saudi Arabia** Hospital distance exceeding 30 minutes (38)		


**Abbreviations:** NYHA: New York Heart Association; HF: heart failure; CABG: coronary artery bypass grafting; ACEIs: angiotensin-converting enzyme inhibitors; ARBs: Angiotensin II receptor blockers.

#### 3.3.1. Social/economic factors

This systematic review found that socioeconomic factors significantly influence medication adherence among HF patients, demonstrating complex associations with education, income, employment, insurance status, and health literacy. Analysis from selected studies indicates a dichotomous effect; higher education, specifically post-secondary, enhances adherence in some cases (32,35,42), while other studies report a negative correlation, linking illiteracy (31) and certain levels of higher education (38) to poorer adherence. Other findings also show decreased adherence with lower education (34,40). Additionally, two studies observed education levels having a neutral impact on adherence (36,39). The effect of income level on adherence has also been mixed, with higher income sometimes correlating with improved adherence (35,40), while in other cases, a higher income was associated with lower adherence (38), or no significant effect was observed (32,34). Employment status varied in impact, from no significant effect (32,34,40) to negative correlations with adherence (38).

Insurance status and health literacy were identified as further determinants of adherence, with insured patients generally exhibiting better adherence (35), while lack of insurance (31) and inadequate health literacy (39) were negatively associated with adherence (36). E-health literacy (36) and familiarity with social media (42) were positively related to adherence.

#### 3.3.2. Patient-related factors

Beginning with age, diverse impacts emerge; younger individuals exhibit elevated adherence (35), while those aged 54 years and above tend to adhere less (42). Nevertheless, multiple studies show no effect of age on adherence (31,32,34,36,38,39,40,41).

Transitioning to gender, discrepancies arise; females exhibit lower adherence rates in some studies (39,40), while others find no significant gender difference on adherence (31,32,35,36,37,38,41,42). Similarly, marital status impacts adherence; married individuals tend to adhere better (35,38), while living alone correlates with poorer adherence in one study (35). However, marital status did not consistently influence adherence across various studies (34,40,42).

BMI, smoking status, and physical activity generally yield neutral effects on adherence (31,34,35,36,38,39,40,42). However, smoking was, in one study, uniquely identified as negatively impacting adherence (31). Housing conditions and family size also influence adherence; urban residence (38) and having fewer than five children (32) are associated with better adherence. Conversely, the habit of khat chewing negatively affects adherence. Lastly, specific biomedical tests, such as blood pressure and heart rate, demonstrate neutral effects on adherence (34,36).

Clinical indicators such as the New York Heart Association (NYHA) classification and ejection fraction do not consistently predict medication adherence. Adherence is observed in Class 1 HF patients and those with ejection fractions between 50 and 60% (42). Yet, adherence decreases with higher NYHA classifications (40) and lower ejection fractions (30–40%) (32). However, some studies have found a significant impact of NYHA classification (34,36) and ejection fraction (34,35,40) on adherence. Additionally, hospitalization history, psychiatric conditions, and sleep disorders influence adherence. Prior hospitalizations can enhance adherence (35), while psychiatric conditions and sleep disorders like depression and insomnia are associated with lower adherence (33,36,41).

#### 3.3.3. Condition factors

The relationship between condition-related factors and medication adherence in HF patients is multifaceted. Duration since diagnosis is a crucial factor; those diagnosed within the first five years generally show better adherence (38,39), while adherence declines in patients diagnosed for over six years (42). However, some studies find no significant impact of disease duration on adherence (34,36). Comorbidities such as mitral valve regurgitation can positively affect adherence (42), whereas diabetes mellitus tends to decrease adherence (35). Temperament factors exhibit a neutral effect on adherence (41). Interestingly, individuals who have undergone coronary artery bypass graft (CABG) surgery tend to demonstrate higher adherence (40).

Clinical indicators like the NYHA classification and ejection fraction are also notable. Higher adherence is observed in Class 1 HF patients and those with ejection fractions between 50 and 60% (42). Yet, adherence decreases with higher NYHA classifications (40) and lower ejection fractions (30–40%) (32). However, some studies have found no significant impact on adherence to NYHA classification (34,36) and ejection fraction (34,35,40). Additionally, hospitalization history, psychiatric conditions, and sleep disorders influence adherence. Prior hospitalizations can enhance adherence (35), while psychiatric conditions and sleep disorders like depression and insomnia are associated with lower adherence (33,36,41).

#### 3.3.4. Medication and institutional factors

The complexity of medication regimens, characterized by the number of medications and specific treatment types, significantly impacts patient adherence. Consuming fewer than five tablets daily and being prescribed angiotensin-converting enzyme (ACE) inhibitors or angiotensin II receptor blockers, along with statin therapy, are associated with higher adherence levels (32,34). In contrast, adherence decreases among patients on anticoagulant medication regimens (34). However, the type or number of medications, daily medication intake, and other medication-related factors such as medication count, dosage intervals, and satisfaction with treatment show the natural effect of adherence (34,36,38,39,40).

The duration of treatment also plays a critical role, with more extended treatment periods associated with lower adherence (38). Adverse drug reactions, particularly side effects, are detrimental to adherence (34). The role of caregiver support appears neutral in influencing adherence (35). Education on HF control, including awareness and health education, shows mixed effects. While understanding HF positively correlates with adherence (42), receiving education, including pharmaceutical health education, has been associated with positive and negative impacts on adherence (38). Moreover, longer distances to the hospital (exceeding 30 minutes) are associated with improved adherence (38).

### 3.4. Assessment of study quality

The CCAT assessments, as detailed in Table 3, revealed a range of study qualities, with scores spanning from 55 to 90%. The analysis highlighted well-articulated objectives, rigorous designs, ethical diligence, and insightful discussions as prevailing strengths across the 12 studies. Despite these strengths, there’s a notable call for better methodological detail, improved sampling, deeper ethical considerations, and more meticulous data handling. Quality classifications using percentiles placed one study (36) in the high-quality bracket (above 88%), five studies in the moderate-quality range (76.5–88%) (34,35,37,40,42), and the remainder as low quality (less than 76.5%) (31,32,33,38,39,41).

**Table 3:** Quality assessment of the included studies.


AUTHORS	PRELIMINARIES	INTRODUCTION	DESIGN	SAMPLING	DATA COLLECTION	ETHICAL MATTERS	RESULTS	DISCUSSION	%	STUDY QUALITY

Lin et al. (2020) (36)	5	5	5	4	5	3	4	5	90%	High

Jarrah et al. (2023) (35)	5	5	4	5	3	4	3	4	83%	Moderate

Shojaee et al. (2023) (42)	4	5	4	4	4	3	5	5	80%	Moderate

Sadeghiazar et al. (2022) (40)	5	5	4	4	3	3	3	4	80%	Moderate

Jarab et al. (2023) (34)	4	5	4	4	4	3	4	4	80%	Moderate

Moaddab et al. (2023) (37)	3	5	4	3	4	4	3	5	78%	Moderate

Rezaei et al. (2022) (39)	4	5	4	4	3	2	3	5	75%	Low

Amininasab et al. (2018) (32)	4	4	4	3	3	4	3	5	75%	Low

Shamsi et al. (2020) (41)	4	4	3	3	3	3	4	4	70%	Low

Raffaa et al. (2020) (38)	4	4	3	1	2	2	3	3	55%	Low

Amininasab et al. (2017) (33)	3	4	3	3	3	4	2	3	63%	Low

Al-Zaazaai et al. 2019 (31)	3	4	2	2	3	2	3	3	55%	Low


## 4. Discussion

While there is a substantial number of systematic reviews available on medication adherence among HF patients worldwide (22,25,26,27), including studies on adherence to treatment for various diseases in the Middle East (43,44,45), this review represents the first thorough examination of presumed rates and factors influencing medication adherence specifically among HF patients in nations within the Middle Eastern region. This review has incorporated 12 primary studies, which collectively encompass 3,122 individuals. Notably, this total includes 600 participants who were analyzed in two studies (32,33). Although both studies utilized the same sample of 300 participants, they are counted separately in the total. Each study provides unique insights into medication adherence, contributing to our understanding without affecting the overall participant total.

The findings of this review underscore a pervasive issue of medication non-adherence among HF patients in several Middle Eastern nations, as elsewhere in the world, mirroring a challenge observed globally. Notably, the adherence rates to medication among these patients exhibit significant fluctuations, ranging from 23.33% to 92.50%. This variability highlights the complex interplay of factors contributing to medication non-adherence, necessitating a closer examination.

At the outset, the substantial range in medication adherence rates captures attention, underscoring the severity and variability of the issue. This sets the stage for a deeper investigation into the myriad influencing factors. These factors encompass socioeconomic elements such as educational attainment, income levels, and insurance status. Individual patient characteristics, including age, gender, and health literacy, also play a critical role. Furthermore, disparities in healthcare provision across different regions, alongside the complexities of medication regimens, exacerbate the variation in adherence rates.

Moreover, the specific aspects of HF under study and differences in patient demographics contribute to the observed discrepancies in medication adherence rates. The challenge of defining and measuring medication adherence introduces additional variability. Discrepancies in methodologies employed to assess compliance, including using different measurement tools, contribute to this issue. Each measurement approach has strengths and limitations, complicating the establishment of a universally accepted standard for assessing medication adherence. Notably, studies that exclusively employed the MMAS questionnaire revealed an average medication non-adherence rate of 57%, highlighting the prevalence of this issue. Addressing the methodological challenges in measuring medication adherence is crucial for understanding the extent of the problem. The variability in definitions and measurement approaches emphasizes the complexity of comprehensively understanding medication non-adherence.

The studies included in this review employed various tools to assess medication adherence, such as the MMAS-4 and MMAS-8. These adherence measurement tools have different structures and scoring systems, which can introduce variability in the reported adherence rates (46). For instance, the MMAS-4 is a shorter version of the MMAS-8, which is more comprehensive and potentially captures a broader range of adherence behaviors (47). This variability in measurement tools can affect the comparability of the results across studies. Studies using different tools may report different adherence rates, which introduces a potential source of bias in the overall findings (48). These discrepancies in adherence measurement should be considered when interpreting the results, as it may influence the consistency and generalizability of the conclusions regarding medication adherence in HF patients (49). To improve future research in this area, it is recommended that standardized adherence measurement tools be used to allow for more accurate comparisons and robust conclusions.

One notable finding emerging from our systematic review is the significant impact of khat chewing on medication adherence among HF patients in Middle Eastern countries, particularly in Yemen. Khat (*Catha edulis*) is a stimulant plant commonly used in East Africa and the Arabian Peninsula. It contains cathinone, which induces effects similar to amphetamines, potentially disrupting medication adherence by impairing sleep and cognitive function and diverting financial resources (50,51,52,53). While this factor has received limited attention in the global literature, Soboka et al. (54) shed light on its relevance in the Ethiopian context, revealing that khat chewing was associated with a higher probability of non-adherence to anti-TB medication. Our study extends this understanding by revealing a similar pattern in Yemen, where khat chewing emerges as a critical factor influencing medication adherence among HF patients. This underscores the importance of considering cultural practices and their effects on healthcare behaviors within specific regional contexts, as khat chewing is widespread in Djibouti, Ethiopia, Kenya, Somalia (including Somaliland), Uganda, and Yemen.

Our analysis highlights how various factors influence medication adherence. While some factors cannot be changed, no single factor alone determines adherence. These factors can change over time and may be both a cause and a consequence of patients not following their medication regimen. It is important not to blame patients for non-adherence alone. Instead, we need to consider broader factors. Social factors, like support networks and financial situations, and healthcare-related factors, such as access to care and how well doctors communicate with patients, play a role. Also, characteristics specific to the condition and how easy it is to use the treatment can affect adherence (55,56,57,58).

Healthcare providers should adopt multifaceted strategies, motivational interviewing, and telephone call interventions to improve medication adherence, mindful of these various influencing factors. Multifaceted strategies involve medication reconciliation, personalized education, collaborative care, and reminders, addressing the complex challenges in adherence (59,60). Motivational interviewing focuses on psychological barriers like lack of motivation, especially in patients with chronic conditions such as HF or those feeling isolated or depressed (61,62). Telephone interventions help overcome socioeconomic and logistical obstacles through regular, personalized support (63,64). These comprehensive interventions tackle both medical, social, emotional, and practical factors, offering a holistic strategy essential for enhancing medication adherence.

The factors influencing medication adherence identified in this systematic review provide insights that are not only relevant to HF patients in the Middle East but may also reflect broader trends observed in other regions. While socioeconomic status, education, and health literacy are commonly recognized determinants of adherence globally, the specific cultural and healthcare contexts in the Middle East may lead to unique manifestations of these factors. Therefore, understanding these localized influences can inform targeted interventions in similar low- and middle-income countries, enhancing the generalizability of findings from this region to other parts of the world.

One of the key strengths of this study is that it is the first systematic review in the Middle East to comprehensively identify and analyze all the factors influencing medication adherence in HF patients. This fills a significant gap in the existing literature, providing valuable insights specific to the region’s unique cultural, socio-economic, and healthcare contexts. Additionally, the study’s use of a broad range of databases and a rigorous quality assessment ensures the findings are robust and relevant. This pioneering work highlights the multifaceted nature of medication adherence in the Middle East. It sets the stage for future research and the development of targeted interventions to improve health outcomes in this population. However, this review has certain limitations. Excluding articles published in languages other than English or Arabic could introduce publication bias. Additionally, excluding Egypt and Israel from the search terms, despite both countries being commonly recognized as part of the Middle East, could also introduce publication bias. This may have resulted in the omission of relevant studies from these countries, potentially affecting the comprehensiveness of our findings. Future research should ensure that all Middle Eastern countries are included to provide a more complete and representative analysis of medication adherence in HF patients across the region. Moreover, while the timeframe for included studies was selected to capture recent advancements, it may inadvertently exclude relevant research from earlier periods. Older studies could offer valuable historical context and trends in medication adherence among HF patients in the Middle East, potentially affecting the comprehensiveness of the review. The small size of study samples constrains the ability to apply the conclusions widely across diverse populations. Most of the studies from Iran may raise the issue of overemphasis on specific healthcare landscapes, cultural factors, and patient behaviors, possibly misrepresenting the experiences of HF patients in other regions of the Middle East. The methodologies employed in these studies, especially using subjective assessments for medication adherence, introduce complexity and potential bias in interpreting the data. Moreover, our analysis did not assess the heterogeneity across the included studies. The quality of the studies reviewed, as evaluated by the CCAT, ranged from low to moderate, highlighting the need for careful consideration of the review’s findings. Many studies suffered from significant methodological shortcomings, including lacking power analysis and ethical concerns, which could increase bias and limit the applicability of the results. Despite thorough quality assessments, these methodological issues and the biases they may introduce emphasize the importance of prudence in interpreting and applying the review’s conclusions. There’s a pressing need for additional studies on medication non-adherence rates and the factors hindering patient adherence. Adopting qualitative or mixed-method research techniques could provide comprehensive insights into the challenges and supports for adhering to medication regimes. Such investigations are vital for determining strategies to boost adherence and improve health outcomes.

## 5. Conclusion

This review underscores the prevalent challenge of medication non-adherence among Middle Eastern HF patients, noting substantial variability in non-adherence rates. This variability could stem from diverse adherence and non-adherence definitions and methodological differences, including the instruments used for measuring adherence. Despite the identification of numerous factors affecting adherence, the review also points to the need for more comprehensive studies in the region, particularly to explore the impact of cultural practices such as khat chewing on medication adherence. Practical strategies to improve adherence include the implementation of community-based educational programs, increasing health literacy through culturally sensitive counseling, and providing consistent follow-up care through pharmacist-led adherence monitoring. Policymakers and healthcare providers should prioritize these actionable steps to overcome barriers to adherence. Future research should aim to elucidate the complex determinants of medication adherence further and develop effective strategies to improve health outcomes for HF patients in the Middle East and beyond.

## Data Accessibility Statement

Datasets used in the research can be obtained from the corresponding author upon reasonable request.

This study is based on a systematic review of previously published literature. No new data were generated or analyzed during this study. All data supporting the findings are available within the included articles and their supplementary materials.

## Additional File

The additional file for this article can be found as follows:

10.5334/gh.1431.s1 Supplementary File. Table S1, Table S2, PRISMA 2020 Main Checklist and PRIMSA Abstract Checklist.
