# Supplementary material for: Medication Adherence and Contributing Factors in Patients with Heart Failure Within the Middle East: A Systematic Review
Source: Glob Heart. 2025 May 27;20(1):47. doi: 10.5334/gh.1431 (PMC12124249; doi:10.5334/gh.1431)
Supplement: Supplementary File. — Table S1, Table S2, PRISMA 2020 Main Checklist and PRIMSA Abstract Checklist. [file gh-20-1-1431-s1.pdf]

Table S1: Searching strategy

Database	Search strategy
**Scopus**	"Medication adherence" OR "Medication compliance" AND "Heart failure" AND "Yemen" OR "Saudi Arabia" OR "Iran" OR "Syria" OR "Jordan" OR "Bahrain" OR "Iraq" OR "Palestine" OR "Kuwait" OR "Lebanon" OR "Emirates" OR "Qatar" OR "Oman"
**PubMed**	(((((((((((((((("Medication Adherence"[Mesh]) AND "Patient Compliance"[Mesh]) AND "Treatment Adherence and Compliance"[Mesh]) AND "Heart Failure"[Mesh]) AND "Middle East"[Mesh]) OR "Yemen"[Mesh]) OR "Jordan"[Mesh]) OR "Saudi Arabia"[Mesh]) OR "Bahrain"[Mesh]) OR "Iran"[Mesh]) OR "Iraq"[Mesh]) OR "Israel"[Mesh]) OR "Kuwait"[Mesh]) OR "Lebanon"[Mesh]) OR "Oman"[Mesh]) OR "Qatar"[Mesh]) OR "Syria"[Mesh]) OR "United Arab Emirates"[Mesh]))
**Google scholar**	Medication adherence OR Medication compliance AND Heart failure AND Yemen OR Saudi Arabia OR Iran OR Syria OR Jordan OR Bahrain OR Iraq OR Palestine OR Kuwait OR Lebanon OR Emirates OR Qatar OR Oman

Table S2: Medication adherence measures used in the included studies

Medication adherence measure (references)	Number of items	Instrument description	Scoring system
**Morisky Medication Adherence Scale (MMAS-8)** [1–4]	8 Items	The Morisky Medication Adherence Scale (MMAS-8) is an eight-item questionnaire designed to assess medication adherence. It is a widely used instrument for evaluating adherence to medications. The MMAS-8 is a meticulously designed self-report evaluation tool aimed at assessing medication adherence. Comprising seven questions answered with a simple yes or no, alongside one question utilizing a five-point Likert scale, it offers a structured approach. The resulting total score spans from 0 to 8, facilitating classification into three distinct levels of adherence: high, medium, and low.	The MMAS-8 offers a comprehensive scoring system spanning from 0 to 8. A score falling below 6 delineates low adherence, while a modest adherence level is reflected by scores of 6 or 7. Achieving a perfect score of 8 signifies a commendable high adherence to medication.
**Morisky Medication Adherence Scale (MMAS-4)** [5–7]	4 Items	The Morisky Medication Adherence Scale (MMAS-4) is utilized to assess patients' medication-taking behavior through a set of four questions. These questions offer binary response options of "yes" or "no" and aim to gauge the consistency of medication intake.	The MMAS-4 scale spans from 0 to 4, it presents a graded assessment of adherence levels. A score of zero signifies robust adherence, while scores of 1 to 2 denote moderate adherence. On the contrary, scores of 3 to 4 signal potential challenges, reflecting lower adherence.
**Medication adherence report scale (MARS-5)** [8,9]	5 Items	The Medication Adherence Report Scale (MARS) is a simple questionnaire with 5 questions. It's used to see how well patients follow their medication plans. Answers are rated on a scale from "most of the times" to "never". The total score can range from 5 to 25, and a higher score means better adherence to medication. This tool found to be reliable, with a score of α = 0.95, and 0.75 respectively in these two studies.	The MARS-5 provides a total score ranging from 0 to 25, wherein scores below 16 indicate low adherence, and a score above 16 indicates high adherence.
**The chronic disease medication adherence questionnaire** [10]	40 items	The chronic disease medication adherence questionnaire is a 40-item 5-point Likert scale type instrument designed in 2012 by Madanloo et al. It has 7 subscales including persistence in treatment, willingness to participate in the treatment, adaptability, incorporating treatment into life, medication adherence, commitment to treatment, and discretion in treatment implementation. In gauging the tool's reliability, the Cronbach's alpha coefficient was applied, yielding values of 0.875 and 0.73 for the questionnaire.	The predetermined minimum and maximum scores for each dimension are transformed into a standardized 0-100 scale, following the guidelines set by the questionnaire designer. The resulting scores classify medication adherence into four levels: poor (0%-25%), moderate (26%-49%), good (50%-74%), and very good (75%-100%).

**Medication adherence measure (references)**	**Number of items**	**Instrument description**	**Scoring system**
**General Medication Adherence Scale (GMAS)** [11]	11 Items	The General Medication Adherence Scale (GMAS) is a tool designed to assess medication adherence. It comprises 11 multiple-choice questions categorized into three domains: patient behavior, pill burden, and additional disease-related expenditures. The scoring system accommodates five levels of adherence, ranging from high to poor. Recently, the questionnaire was validated in one 3rabic country with chronic conditions, demonstrating strong reliability (Cronbach's of 0.865).	The scoring criteria encompass a range of adherence levels, which are delineated as follows: high adherence (scores between 30 and 33), good adherence (scores between 27 and 29), partial adherence (scores between 17 and 26), low adherence (scores between 11 and 16), and poor adherence (scores of 10 or lower).
**The Medication Adherence Report Scale (MARS-10)** [12]	10 Items	The MARS-10 is a questionnaire used to understand how well someone sticks to their medication routine over the past week. It has ten questions with "Yes" or "No" answers. Scores range from 0 to 10, where higher scores mean better adherence. Those scoring 0 to 5 are labeled as 'non-compliant', while those scoring 6 to 10 are 'compliant'. In this study, it was used to see how well people with heart failure followed their medication plans.	Scores 0 to 5 reflects non-compliant, while score 6 to 10 reflects compliant.

PRISMA 2020 Main Checklist

Topic	No.	Item	Location where item is reported
**TITLE**			
**Title**	1	Identify the report as a systematic review.	Title page
**ABSTRACT**			
**Abstract**	2	See the PRISMA 2020 for Abstracts checklist	
**INTRODUCTION**			
**Rationale**	3	Describe the rationale for the review in the context of existing knowledge.	Introduction
**Objectives**	4	Provide an explicit statement of the objective(s) or question(s) the review addresses.	Introduction
**METHODS**			
**Eligibility criteria**	5	Specify the inclusion and exclusion criteria for the review and how studies were grouped for the syntheses.	Study Selection and Eligibility Requirements
**Information sources**	6	Specify all databases, registers, websites, organisations, reference lists and other sources searched or consulted to identify studies. Specify the date when each source was last searched or consulted.	Data Search Strategy
**Search strategy**	7	Present the full search strategies for all databases, registers and websites, including any filters and limits used.	Data Search Strategy & Supplementary Materials Table S1
**Selection process**	8	Specify the methods used to decide whether a study met the inclusion criteria of the review, including how many reviewers screened each record and each report retrieved, whether they worked independently, and if applicable, details of automation tools used in the process.	Study Selection and Eligibility Requirements
**Data collection process**	9	Specify the methods used to collect data from reports, including how many reviewers collected data from each report, whether they worked independently, any processes for obtaining or confirming data from study investigators, and if applicable, details of automation tools used in the process.	Study Selection and Eligibility Requirements
**Data items**	10a	List and define all outcomes for which data were sought. Specify whether all results that were compatible with each outcome domain in each study were sought (e.g. for all measures, time points, analyses), and if not, the methods used to decide which results to collect.	Outcome measures

Topic	No.	Item	Location where item is reported
	10b	List and define all other variables for which data were sought (e.g. participant and intervention characteristics, funding sources). Describe any assumptions made about any missing or unclear information.	Outcome measures
**Study risk of bias assessment**	11	Specify the methods used to assess risk of bias in the included studies, including details of the tool(s) used, how many reviewers assessed each study and whether they worked independently, and if applicable, details of automation tools used in the process.	Quality Evaluation
**Effect measures**	12	Specify for each outcome the effect measure(s) (e.g. risk ratio, mean difference) used in the synthesis or presentation of results.	Data Synthesis and Analysis
**Synthesis methods**	13a	Describe the processes used to decide which studies were eligible for each synthesis (e.g. tabulating the study intervention characteristics and comparing against the planned groups for each synthesis (item 5)).	Study Selection and Eligibility Requirements
	13b	Describe any methods required to prepare the data for presentation or synthesis, such as handling of missing summary statistics, or data conversions.	Data Synthesis and Analysis
	13c	Describe any methods used to tabulate or visually display results of individual studies and syntheses.	Data Synthesis and Analysis
	13d	Describe any methods used to synthesize results and provide a rationale for the choice(s). If meta-analysis was performed, describe the model(s), method(s) to identify the presence and extent of statistical heterogeneity, and software package(s) used.	Data Synthesis and Analysis
	13e	Describe any methods used to explore possible causes of heterogeneity among study results (e.g. subgroup analysis, meta-regression).	Not applicable
	13f	Describe any sensitivity analyses conducted to assess robustness of the synthesized results.	Not applicable
**Reporting bias assessment**	14	Describe any methods used to assess risk of bias due to missing results in a synthesis (arising from reporting biases).	Quality Evaluation
**Certainty assessment**	15	Describe any methods used to assess certainty (or confidence) in the body of evidence for an outcome.	Not applicable
**RESULTS**			
**Study selection**	16a	Describe the results of the search and selection process, from the number of records identified in the search to the number of studies included in the review, ideally using a flow diagram.	Figure 1
	16b	Cite studies that might appear to meet the inclusion criteria, but which were excluded, and explain why they were excluded.	Figure 1

Topic	No.	Item	Location where item is reported
**Study characteristics**	17	Cite each included study and present its characteristics.	Table 1
**Risk of bias in studies**	18	Present assessments of risk of bias for each included study.	Table 3
**Results of individual studies**	19	For all outcomes, present, for each study: (a) summary statistics for each group (where appropriate) and (b) an effect estimate and its precision (e.g. confidence/credible interval), ideally using structured tables or plots.	Geographical Patterns and Patient Demographics
**Results of syntheses**	20a	For each synthesis, briefly summarise the characteristics and risk of bias among contributing studies.	Assessment of Study Quality
	20b	Present results of all statistical syntheses conducted. If meta-analysis was done, present for each the summary estimate and its precision (e.g. confidence/credible interval) and measures of statistical heterogeneity. If comparing groups, describe the direction of the effect.	Table 2
	20c	Present results of all investigations of possible causes of heterogeneity among study results.	Not applicable
	20d	Present results of all sensitivity analyses conducted to assess the robustness of the synthesized results.	Not applicable
**Reporting biases**	21	Present assessments of risk of bias due to missing results (arising from reporting biases) for each synthesis assessed.	Assessment of Study Quality
**Certainty of evidence**	22	Present assessments of certainty (or confidence) in the body of evidence for each outcome assessed.	Not applicable
**DISCUSSION**			
**Discussion**	23a	Provide a general interpretation of the results in the context of other evidence.	Discussion
	23b	Discuss any limitations of the evidence included in the review.	Discussion
	23c	Discuss any limitations of the review processes used.	Discussion
	23d	Discuss implications of the results for practice, policy, and future research.	Discussion
**OTHER INFORMATION**			
**Registration and protocol**	24a	Provide registration information for the review, including register name and registration number, or state that the review was not registered.	Not applicable
	24b	Indicate where the review protocol can be accessed, or state that a protocol was not prepared.	Not applicable
	24c	Describe and explain any amendments to information provided at registration or in the protocol.	Not applicable

Topic	No.	Item	Location where item is reported
**Support**	25	Describe sources of financial or non-financial support for the review, and the role of the funders or sponsors in the review.	Funding
**Competing interests**	26	Declare any competing interests of review authors.	Conflict of interest
**Availability of data, code and other materials**	27	Report which of the following are publicly available and where they can be found: template data collection forms; data extracted from included studies; data used for all analyses; analytic code; any other materials used in the review.	Data availability

PRIMSA Abstract Checklist

Topic	No.	Item	Reported?
**TITLE**			
**Title**	1	Identify the report as a systematic review.	Yes
**BACKGROUND**			
**Objectives**	2	Provide an explicit statement of the main objective(s) or question(s) the review addresses.	Yes
**METHODS**			
**Eligibility criteria**	3	Specify the inclusion and exclusion criteria for the review.	Yes
**Information sources**	4	Specify the information sources (e.g. databases, registers) used to identify studies and the date when each was last searched.	Yes
**Risk of bias**	5	Specify the methods used to assess risk of bias in the included studies.	Yes
**Synthesis of results**	6	Specify the methods used to present and synthesize results.	Yes
**RESULTS**			
**Included studies**	7	Give the total number of included studies and participants and summarize relevant characteristics of studies.	Yes
**Synthesis of results**	8	Present results for main outcomes, preferably indicating the number of included studies and participants for each. If meta-analysis was done, report the summary estimate and confidence/credible interval. If comparing groups, indicate the direction of the effect (i.e. which group is favoured).	Yes
**DISCUSSION**			
**Limitations of evidence**	9	Provide a brief summary of the limitations of the evidence included in the review (e.g. study risk of bias, inconsistency and imprecision).	Yes
**Interpretation**	10	Provide a general interpretation of the results and important implications.	Yes
**OTHER**			
**Funding**	11	Specify the primary source of funding for the review.	No
**Registration**	12	Provide the register name and registration number.	No
